# Dissociative Experiences and Substance Use Disorder in Adulthood After Childhood Trauma: A Systematic Review of the Literature

**DOI:** 10.32872/cpe.15877

**Published:** 2025-11-28

**Authors:** Cory Julien, Laura Bernard, Vincent Brejard

**Affiliations:** 1 Department of Clinical Psychology, Aix-Marseille University, LPCPP, UR 3278, Aix-en-Provence, France; University of Fribourg, Fribourg, Switzerland

**Keywords:** childhood trauma, C-PTSD, dissociation, substance use disorder, adults

## Abstract

**Context:**

Childhood trauma is more prevalent among individuals with substance use disorders compared to the general population, representing a significant public health concern. The presence of comorbid dissociative symptoms poses a significant challenge for psychological care.

**Objectives:**

We conducted a systematic review of the literature, using the PRISMA method, to establish the relationship between dissociative experiences and substance misuse in adults who have experienced traumatic childhood events.

**Method:**

We used electronic databases (PubMed, PsycInfo, PsycArticles, Web of Science and ProQuest) up to August 2023. Studies were selected which included adults over 18 years old who had been exposed to one or more traumatic events in childhood, and which jointly assessed Substance Use Disorder (SUD) and dissociation, using quantitative methodology. The review included both cross-sectional and longitudinal studies, with the risk of bias assessed using the AXIS tool and the Qualitative Assessment Tool for Observational Cohort and Cross-Sectional Studies. The results are entered in a table and analyzed using a narrative summary.

**Results:**

Among the 18 included studies, encompassing a total of 6,451 participants, the majority (*n* = 10) showed a significant positive correlation between dissociative experiences and SUD. The studies collectively indicate a general trend: childhood traumatic antecedents can influence the severity of dissociative symptomatology and SUD.

**Discussion:**

These results are discussed in greater depth in relation to the two main theories explaining the link between SUD and dissociation, namely *self-medication* and *chemical dissociation theory.* This paper clarifies the relationship between dissociation and substance use in a population traumatized in childhood, although the heterogeneity of the studies necessitates a cautious interpretation of this primary finding.

## Highlights

- Early traumatic experiences are a high-risk factor for substance use disorder in adulthood.
- The more frequent the dissociative experiences are, the more severe the substance use tends to be.
- Substance abuse in individuals with traumatic childhood could be explained by chemical dissociation theory.

## Context

Childhood trauma is more prevalent among individuals with substance use disorders compared to the general population (Garami et al., 2019; Scheidell et al., 2018). The cumulative nature of childhood traumatic experiences and their impact on the severity of traumatic symptoms and the onset of substance use disorders represents a significant public health concern (Zhang et al., 2020).

Exposure to verbal, physical or sexual abuse or neglect during childhood can lead to the subsequent development of physiological, psychological and neurological disorders in adulthood (Dye, 2018; Gupta, 2013; Kratzer et al., 2022). The Adverse Childhood Experience study (Felitti et al., 1998) has already highlighted the impact of stressful childhood experiences on the adult lives of these individuals. This population generally presents more comorbidities in adulthood, including addiction, depression, suicide attempts, endangerment and somatic illnesses (Felitti et al., 1998; Rogerson et al., 2023), which can lead to premature death. Children who have experienced sexual trauma are the population with the most post-traumatic symptoms (study conducted on children and adolescents aged between 8 and 17, Lofthouse et al., 2024). These symptoms, identified by the diagnostic term Post-Traumatic Stress Disorder (PTSD) as described in the DSM-5, include intrusion, persistent avoidance associated with the event, alterations in cognition and mood, and changes in arousal persist for more than a month (American Psychiatric Association, 2013). When exposure to the event is prolonged or repetitive, as is often the case with childhood traumatic experiences, other disorders may emerge involving all the criteria of PTSD just cited, but also affect/emotion regulation disorders, a sense of being diminished, coupled with feelings of shame and guilt, as well as difficulties maintaining relationships (WHO, 2019): this condition is referred to as Complex Post-Traumatic Stress Disorder (C-PTSD).

Individuals who experience potentially traumatic events in childhood, such as those previously mentioned, are more likely to present high levels of dissociative symptoms (Fung et al., 2023; Gobin & Freyd, 2017). This dissociative symptomatology can manifest as depersonalization, derealization and partial or total traumatic amnesia (Nijenhuis et al., 1996). The traumatic model of dissociation (Dalenberg & Carlson, 2012) suggests that it functions as an adaptative mechanism, according to Pavlov's classical conditioning theory from 1903 (Lam et al., 2024). Dissociation is considered as a defense mechanism occurring preconsciously (Kennedy et al., 2004). Thus, dissociation, initially experienced during early traumatic experiences, continues to be used as an emotional self-regulation strategy in response to intense emotions (Lam et al., 2024; Smith, 2021). Research on the psychopathological interactions between dissociative mechanisms and trauma is ongoing and expanding in the literature. However, when this comorbidity is present, aggravated symptomatology is clinically observed: greater symptoms of reliving (Burton et al., 2018), high rates of psychiatric comorbidities (Herzog et al., 2020), feelings of personal devaluation and diminished well-being (Bateman et al., 2024), higher levels of substance use (Thal et al., 2019). However, the presence of the dissociative mechanism predicts an unfavorable prognosis for the improvement of traumatic symptomatology during its management (Ginzburg et al., 2006).

Various theories have attempted to explain the comorbid onset of substance use, such as biopsychosocial or biomedical models, which conceptualize addiction in its multifaceted nature (Skewes & Gonzalez, 2013; Volkow et al., 2016). In clinical psychopathology, Khantzian (1997) theorized the *self-medication theory*. Substances are used to regulate negative affects that cannot be regulated by the individual and his or her personal resources. This theory has been further developed by other authors, who hypothesize that the psychotropic effects of substances could regulate negative post-traumatic affects, that individuals are unable to control (Bordieri et al., 2014; Kearns et al., 2019; Simpson et al., 2014). Each substance appears to regulate specific behaviors or affects in individuals with emotional regulation problems and maladaptive behaviors: opiates would seem to act on intense and violent affects, alcohol would reduce depressive symptoms such as isolation, and stimulants would reduce hyperactivity. However, this theory does not account for dissociative experiences.

Another hypothesis is proposed by Somer et al. (2010), entitled the *chemical dissociation theory*, which posits that the effects of substances on psychological functioning enable the maintenance of the dissociative mechanism. Somer's original study focuses on opioid use as a coping strategy when individual regulatory strategies are insufficient. Consequently, dissociative experiences stemming from childhood trauma appear to be perpetuated into adulthood through the use of various drugs or alcohol (Romano, 2015). Early traumatic experiences therefore represent a risk factor for several comorbidities in adulthood, notably SUD (Blanco et al., 2020).

The frequent co-occurrence of these three clinical disorders – traumatic childhood experiences, dissociation and SUD – complicates psychotherapeutic management (Bellet & Varescon, 2019; Camille, 2022). While previous research has extensively explored the comorbidity between PTSD and SUD, the specific role of dissociative experiences in this relationship, particularly in individuals with a history of childhood trauma, remains unclear. To our knowledge, no systematic review has synthesized the evidence addressing this triad. The aim of this systematic review is to identify the links between dissociative experiences and SUD in adults who have experienced childhood trauma. Confirming these links is crucial for optimizing therapeutic care. The therapeutic management of these two concomitant disorders is still being debated in the literature, particularly regarding sequential or integrated treatment. The recent meta-analysis by Hien et al. (2024) has demonstrated that SUD and PTSD can be treated simultaneously (Baker et al., 2012), leading us to question the place of dissociative symptomatology. Incorporating dissociation into the treatment of these two disorders may prove beneficial if this review supports the existence of a relationship.

## Method

### Search Strategy

The study followed the Preferred Reporting Items for Systematic Reviews and Meta-Analyses (PRISMA) guidelines and a PRISMA flow chart (Page et al., 2021) The PRISMA method facilitates the writing and reading of systematic literature reviews and meta-analyses by providing 27 methodological points to follow. Our article inclusion strategy followed the PICOTS framework. The excel of eligible articles is publicly available at the OSF (Julien et al., 2024S).

The bibliographic search was conducted on the following databases: PubMed, PsycInfo, PsycArticles, Web of Science and ProQuest, with the following keywords defined according to the Medical Subject Headings (MeSH): (substance-related disorders OR chemical dependence OR drug abuse OR drug use disorders OR substance addiction OR substance use OR substance use disorders) AND (post traumatic stress disorder OR posttraumatic stress disorder OR PTSD OR moral injury OR chronic post traumatic stress disorder OR trauma) AND (dissociative disorders OR dissociation OR dissociative reactions). Filters were: "English", "2014-2023", "adults", "articles". These keywords and filters were applied across the five databases mentioned, following the PRISMA method. The complete search chain comprises all these elements for each database.

We opted to use keywords related to PTSD rather than childhood aversive experiences based on tests conducted in our databases. Using PTSD-related keywords allowed us to access studies that consider childhood aversive experiences, which are often mentioned in the abstract rather than the title.

### Study Selection

#### Inclusion and Exclusion Criteria

Studies were included if they: (a) assessed adults who have experienced a traumatic event in childhood, (b) jointly assessed the variables childhood trauma, SUD and dissociation, (c) presented comparisons with control groups, community samples as well as inpatient and outpatient clinical samples, (d) had a quantitative methodology, (e) were published in English.

Manuscripts and doctoral theses were excluded. The period of publication of these articles was between January 2014 and March 2023. This time frame was selected based on an assessment of the number of publications on psychological trauma in Pubmed. The number of publications increases significantly from 2014 onwards, as indicated by the ‘results by year’ graph. The 2014 limit also allows us to concentrate on the last ten years in order to report on the most recent research on the subject.

Studies were excluded if they: (a) did not indicate the period in which trauma events were experienced (the traumatic event must have occurred in childhood), (b) dealt with the dissociative identity disorder, (c) dealt with behavioral addictions, (d) studied the effects of alcoholization in experimental situations, (e) reported on methadone substitution treatment.

#### Screening Process

Articles were transferred to a Zotero (2019) folder when the title appeared to meet our objective. Utilizing its Google Chrome extension, Zotero enabled us to save all relevant articles directly into a dedicated folder, facilitating quick access, sorting according to our inclusion and exclusion criteria by creating different sub-folders, and better identification of duplicates. Subsequently, selection was performed based on abstracts. To be selected, abstracts had to present a population that had experienced childhood trauma and at least mention our two target variables, i.e., dissociation and substance misuse. Finally, the articles retained were read in their entirety, refining the final selection stage.

**Figure 1:**
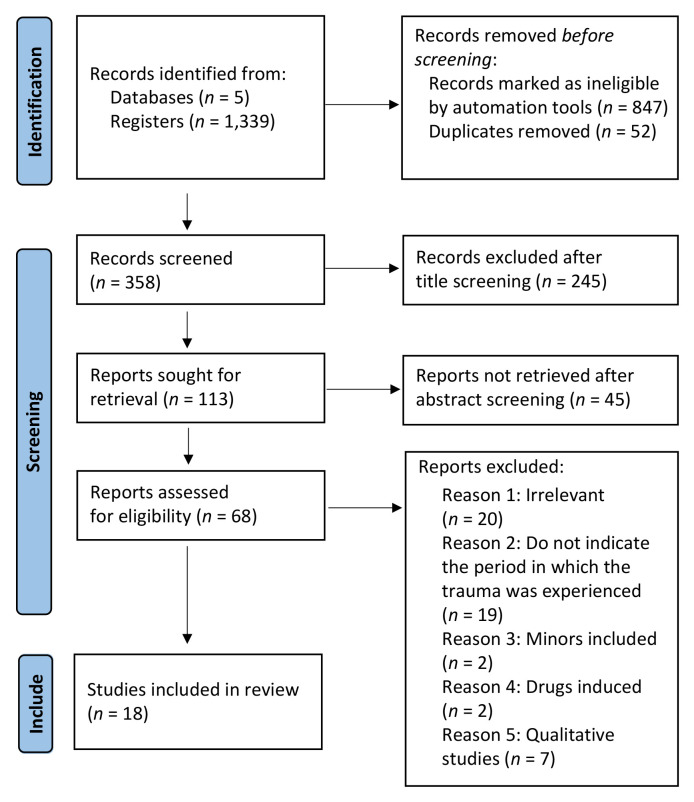
Flowchart of Search Strategy

Two investigators (CJ and LB) carried out this selection independently. Additionally, a bibliographic monitoring was established from the screening process to keep authors updated with new publications on the subject. Each data base was last consulted in August 2023. The third investigator was called upon in the event of disagreement (VB). The publication search and search strategy are presented in a flowchart (Figure 1).

#### Data Extraction

The investigators developed a standardized data extraction file to compile all the essential information from the included publications: authors and year of publication, study location, sample characteristics, methodology used and main results relevant to our objective (Table 1). One investigator (CJ) extracted the data, which were then independently verified by another investigator (VB). The selected results pertained to the link between dissociative experiences and substance use and were documented in an Excel file.

#### Study Risk of Bias Assessment

**Figure 2:**
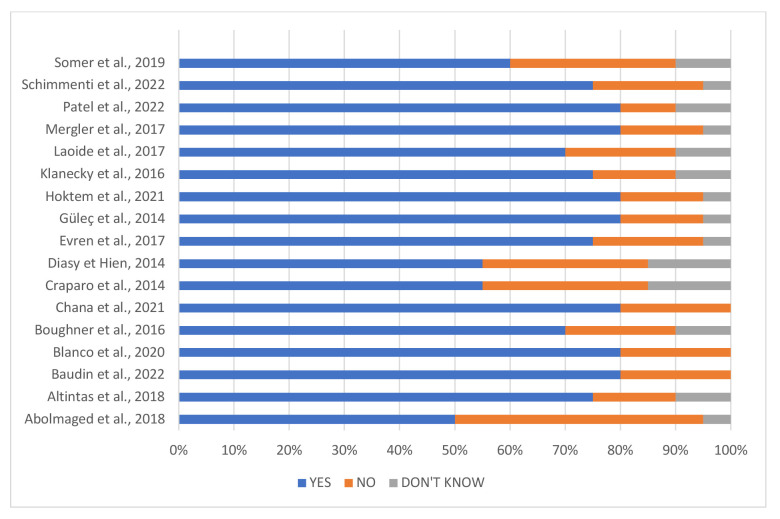
Results of Studies’ Limits According to the AXIS Tool for Cross-Sectional Design Risk of Bias

**Figure 3:**
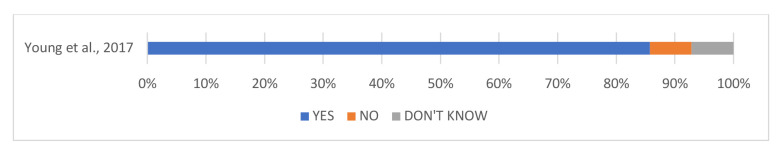
Results of Study’ Limits According to the Qualitative Assessment Tool Observational Cohort and Cross-Sectional Studies

The risk of bias inherent in each study was measured using the AXIS questionnaire for cross-sectional studies (*n* = 17; Downes et al., 2016) and the Quality-Assessment-Tool-for-Observational-Cohort-and-Cross-Sectionnal-Studies questionnaire for longitudinal study (*n* = 1). AXIS and Quality-Assessment-Tool questionnaires assessed the quality of studies through 20 and 12 questions respectively, which can be answered with “yes”, “no” or “I don't know”, on the clarity of objectives, method, results and discussion. Results are presented in Figures 2 and 3. The principal investigator carried out this risk of bias assessment (CJ) and check by another researcher (VB).

## Results

### Characteristics of Included Studies

A total of 1,339 studies were identified by the five databases mentioned. 52 duplicate articles were removed, and only 358 articles were retained after filtering. The first filtering by title revealed 113 eligible articles; the second after reading of abstracts admitted 68 eligible articles.

A total of 18 studies were finally included in this systematic literature review (for total of 6,451 participants): after full reading, some articles were found to be off-topic (*n* = 20), others to have no specification of the period in which trauma events were experienced (*n* = 19), to have included minors (*n* = 2), to have admitted researcher-induced substance use (*n* = 2), or to have been qualitative studies (*n* = 7). These results are presented in Table 1.

**Table 1:** Included Studies

Author/Year	Country	Sample			Methods/Measurements		Results
		Groups	*M* (*SD*) Age	Gender	Questionnaires	Time paradigm	
1. Abolmaged et al., 2018	Egypt	Group A: Patients BPD (*n* = 40) Group B: Patients BDP + SUD (*n* = 40)	24.55 (6.77) 31.30 (11.01)	100% of women	SCID I SCID II BPDSI-IV CTQ DES	Single session	Group A = dissociative experiences greater than Group B.
2. Altintas & Bilici, 2018	Turkey	Prison inmates (*n* = 200)	18-65 years	50% of women	Interviews CTQ-28 DES	Single session	No direct link between SUD and dissociation.
3. Baudin et al., 2022	France	Patients hospitalized in rehabilitation centers (*n* = 568)	44.5 (9.0)	15.3% of women	CTQ DES AUD BDI STAI PCL-5	Single session two weeks after detox period	Subjects with high dissociation + SUD = more severe SUD, depression, anxiety and traumatic symptoms, more childhood trauma.
4. Blanco et al., 2020	Spain	Hospitalized patients (*n* = 150) Dual disorder *n* = 100 Only SUD *n* = 50	44 (10)	38% of women	SDS EGEP-5 CTQ DES The Holmes-Rahe Life Stress Inventory DDSI PRISM HDRS YMRS BPRS	Single session after initial detox period	No significant correlation between SUD and dissociation.
5. Boughner & Frewen, 2016	USA	Group A: Online patients (*n* = 513) Group B: Clinical sample (*n* = 12) Total *n* = 525	34.39 (11.18)	54% of women (Group A) 98% of men (Group B)	LEC-5 ACE PCL-5 SSI-SA PCR Scaling ASSIST	Single session	Correlation SSI-SA – dissociation: Online sample (females): *r* = .178; *p* < .01 Online sample (males): *r* = .271; *p* < .01 Clinical sample (males): *r* = -.021; ns
6. Chana et al., 2021	USA	Women prisoners (*n* = 508)	42.33 (11.71)	100% of women	Betrayal trauma Trauma Symptoms Checklist 40 AUDIT DAST-10	Single session	AUD – Dissociation: *r* = .02; ns SUD – Dissociation: *r* = .21; *p* < .01
7. Craparo et al., 2014	Italy	Outpatients program for alcohol-dependence (*n* = 234) Control group (*n* = 117)	Target group = 44.85 (9.94) Control group = 43.98 (9.57)	60 men and 57 women in each group	DES-II TAS-20 TEC	Single session	Group AUD: Correlation childhood trauma - dissociation *r* = .22; *p* < .05 Control group: *r* = .39; *p* < .01
8. Daisy & Hien, 2014	USA	Inner city women (*n* = 148)	32.8 (7.9)	100% of women	Demographic and Treatment History Form SCID CTQ Childhood Sexual Abuse Interview CTS-Partner DES	Single session	Correlation SUD – dissociation Standardized beta = .34; *p* < .001 => *r* = .84
9. Evren et al., 2017	Turkey	Inpatients with substance use disorder (*n* = 190): Group 1: no risk (*n* = 51) Group 2: low risk (*n* = 86) Group 3: high risk (*n* = 53)	45.18 (10.68) 44.35 (10.41) 44.77 (9.51)	100% of men	BDI ASRS CTQ-28 DES	Single session 3-4 weeks after the last day of drinking	Correlation AUD – dissociation: *r*^2^ = .374; *p* < .001 => *r* = .612
10. Güleç et al., 2014	Turkey	Inpatients with conversion disorder (*n* = 94): Group 1: suicide attempts (+; SA) *n* = 33 Group 2: Suicide attempts (-; nSA) *n* = 61 Healthy control (*n* = 50)	30.33 (10.71) 30.82 (10.83) 34.64 (11.94)	85% of women	SCID-I BDI BAI TAS-20 CTQ-28 DES TCI	Single session	No direct link between SUD and dissociation.
11. Hoktem et al., 2021	Turkey	University student (*n* = 300)	Group 18-22 years and group 22-25 years	145 women on 300	CTQ DES AUDIT DUDIT	Single session	Correlation AUDIT-DES: *r* = .486; *p* < .01 Correlation DUDIT-DES: *r* = .552; *p* < .01
12. Klanecky et al., 2016	USA	University students (*n* = 213)	19.56 (1.12)	63.4% of men	AUDIT DERS DES-II ETI-SR-SF PCL-C	Single session	Correlation AUDIT-DES: *r* = .368; *p* < .01
13. Ó Laoide et al., 2018	Irland	Media and press participants (*n* = 761)	21.46 (2.45)	69.6% of women	CTQ CDS ECR-RS AEE DASS-21	Single session	Drugs and detection of DP OR = 0.90 → *r* = -.029; ns
14. Mergler et al., 2017	Germany	Inpatients (*n* = 337) and outpatients (*n* = 122) from addiction centers	36.7 (11.2)	59.7% of men	IDCL PDS DES CTQ EuropASI	Single session	Association of the two groups around the SUD: OR = 0.878; ns → *r* = -.036
15. Patel et al., 2022	Canada	334 inpatients care for PTSD	44.29 (9.77)	50% of women	PCL-5 MDI AUDIT DERS ACES	Single session	Dissociation – Alcohol related problem: *r* = .184; *p* < .05
16. Schimmenti et al., 2022	Italy	1040 community-dwelling adults	29.55 (11.37)	67% of women	CTQ-SF DES-T Level2-Substance Use-Adult	Single session	Correlation pathological dissociation – severity of substance use: *r* = .36; *p* < .001
17. Somer et al., 2019	Israel	Recovering patients R-SUD (*n* = 100) Control group (*n* = 80)	40.2 41.9	100% of men	CTQ DES MDS-16	Single session	R-SUD and dissociation: *d* = 0.50; *p* < .001 → *r* = .243
18. Young et al., 2017	USA	Homeless or unstably housed women (*n* = 300)	46.99 (8.62)	100% of women	Severity of Violence Against Women Scales DES DIS	One session every 6 months for 3 years (7 time points)	No direct link between SUD and dissociation.

*Note*. **BPD**: borderline personality disorder; **SUD**: substance use disorder; **R-SUD**: Recovering from Substance Use Disorder; **DP**: Depersonalization; **AUD**: Alcohol use disorder; **SA**: suicide attempts. **Questionnaires: SCID**: Structured clinical interview for DSM-IV (Axis I and II); **BPDSI**: Borderline personality disorder severity index; **CTQ**: Childhood traumatic questionnaire; **DES**: Dissociative experience scale; **BDI**: Beck depression inventory; **STAI**: State-Trait anxiety inventory; **PCL**: PTSD checklist; **SDS**: Severity of dependance scale; **EGEP**: Global assessment of Posttraumatic Stress Questionnaire; **DDSI**: Dual diagnostic screening interview; **PRISM**: Psychiatric Research Interview for Substance and Mental Disorders; **HDRS**: Hamilton Depression Rating Scale; **YMRS**: Young Mania Rating Scale; **BPRS**: Brief Psychiatric Rating Scale; **LEC**: Lifetime Events Checklist; **ACE**: Adverse Childhood Experience; **SSI-SA**: Simple Screening Instrument for Substance Abuse; **PCR**: Perceived Causal Relations; **ASSIST**: Alcohol, Smoking, and Substance Involvement Screening Test; **AUDIT / DUDIT**: Alcohol / Drug Use Disorder Identification Test; **DAST**: Drug Abuse Screening Test; **TAS**: Toronto Alexithymia Scale; **TEC**: Traumatic Experience Checklist; **CTS-Partner**: Conflict Tactics Scale-Partner Version; **ASRS**: Adult attention-deficit/hyperactivity disorder Self-Report Scale; **BAI**: Beck Anxiety Inventory; **TCI**: Temperament and Character Inventory; **DERS**: Difficulties in Emotion Regulation Scale; **ETI-SR-SF**: Early Trauma Inventory – Self Report – Short Form; **CDS**: Cambridge Depersonalization Scale; **ECR-RS**: Experiences in Close Relationships – Relationship structures questionnaire; **AEE**: Attitudes toward Emotional Expression; **DASS**: Depression, Anxiety and Stress Scale; **IDCL**: International Diagnostic Checklist; **PDS**: Posttraumatic Diagnostic Scale; **ASI**: Addiction Severity Index; **MDI**: Multiscale Dissociation Inventory; **ACES**: Adverse Childhood Experience Scale; **MDS**: Maladaptive Daydreaming Scale; **DIS**: Diagnostic Interview Schedule.

The 18 included studies were all published between 2014 and 2022, conducted in Canada, Egypt, France, Germany, Ireland, Israel, Italy, Spain, Turkey and the USA. Sample heterogeneity was observed, encompassing incarcerated individuals, inpatients or outpatients from addictology and/or psychotraumatology units, students, community or homeless people. Psychiatric diagnoses also varied (borderline personality, mood disorder, SUD, dual disorder, etc.).

The studies featured an almost equal ratio of men to women (55% women to 45% men). Half of the included studies (*n* = 9) focused on a specific sample, while the other half (*n* = 9) made a group comparison, between subgroups or using a control group. The studies were mainly cross-sectional (*n* = 17), with only the Young et al. (2017) being longitudinal, with a session every six months for three years focusing on homeless or unstably housed women.

### Assessment Tools Used

Dissociative symptoms were assessed in the majority of studies (*n* = 13) by the Dissociative Experience Scale (DES), focused on various dissociative events in the participant's daily life. One study used another version of the DES, the Dissociative Experience Scale-Taxon (DES-T; Schimmenti et al., 2022), including so-called pathological dissociation. The remaining five studies used the Multiscale Dissociation Inventory (MDI; Patel et al., 2022), the PTSD Checklist for DSM-5 (PCL-5; Boughner & Frewen, 2016), the Cambridge Depersonalization Scale (CDS; Ó Laoide et al., 2018), or the Trauma Symptom Checklist-40 (TSC; Chana et al., 2021).

Childhood traumas were assessed by the Childhood Trauma Questionnaire (CTQ) in most studies (*n* = 12), including emotional, physical and sexual abuse, as well as physical and emotional neglect. The remaining studies used the Adverse Childhood Experiences questionnaire (ACE; Boughner & Frewen, 2016; Patel et al., 2022); the Traumatic Experiences Checklist (TEC; Craparo et al., 2014); the Early Trauma Inventory – Self Report – Short Form (ETI-SR-SF; Klanecky et al., 2016); the Severity of Violence Against Women Scale (Young et al., 2017); and the Childhood Experiences of Violence Questionnaire combined with questions chosen and created by the authors (Chana et al., 2021).

Finally, substance use was generally identified by self-report and/or urine testing (*n* = 8). Five studies used the Alcohol Use Disorder Test (AUDIT; Baudin et al., 2022; Chana et al., 2021; Hoktem et al., 2021; Klanecky et al., 2016; Patel et al., 2022). The study by Hoktem et al. (2021) also combined the AUDIT with the Drug Use Disorder Identification Test (DUDIT), and another study, Chana et al. (2021) combined the AUDIT with the Drug Abuse Screening Test (DAST). The other studies’ assessment tools were more heterogeneous: the Severity of Dependence Scale (SDS; Blanco et al., 2020); the Simple Screening Instrument for Substance Abuse (SSI-SA; Boughner & Frewen, 2016); and the Level 2-Substance Use-Adult (Schimmenti et al., 2022). Two studies assessed substance use disorders with a semi-structured interview: the Structured Clinical Interview for DSM-IV (SCID; Daisy & Hien, 2014) and the European Addiction Severity Index (EuropASI; Mergler et al., 2017).

The substances covered in these articles, listed in six studies, include hallucinogens or disruptors (such as cannabis and ecstasy), depressants (heroin), stimulants (amphetamines, cocaine, methamphetamine), inhalants, opiates and opioids, sedatives (such as benzodiazepines) and nicotine.

### Correlations Among Dissociation, SUD and Traumatic Events in Childhood

Of the 18 studies identified for this review, only 12 provided quantitative data on the link between dissociation and substance use disorder in adults with childhood psychotrauma. Due to considerable heterogeneity among the studies, a meta-analysis could not be conducted, as it was uncorrected by various moderators tested (e.g. socio-demographic data, clinical or non-clinical population).

Only the three studies by Ó Laoide et al. (2018), Mergler et al. (2017), and the male clinical subgroup of the Boughner and Frewen (2016) study showed a negative correlation between dissociation and substance misuse in their respective study populations. These correlations (respectively *r* = -0.029, *r* = -0.036 and *r* = -0.021) were, however, very weak, non-significant with a negligible effect size according to Cohen's criteria.

Significant positive correlation coefficients were found in 10 studies. Only two subgroups did not have significant effect sizes: the alcohol subgroup in Chana et al. (2021) study and the male clinical subgroup in Boughner and Frewen (2016) study. Thus, effect sizes were greater in studies highlighting positive correlations, with correlation coefficients ranging from *r* = 0.178; *p* < .01 (Boughner & Frewen, 2016) to *r* = 0.552; *p* < .01 (Hoktem et al., 2021).

For the remaining studies, the authors documented direct links between traumatic events in childhood and dissociative experiences and/or traumatic events in childhood and SUD, without specifically highlighting a direct link between dissociation and SUD (Altintas & Bilici, 2018; Güleç et al., 2014; Young et al., 2017). Blanco et al. (2020) acknowledged that the correlation between dissociation and SUD was not significant, without mentioning its value. Abolmaged et al. (2018) observed that participants with borderline personality disorder and SUD had fewer dissociative experiences than the group with substance use disorder only. The authors attempted to explain this outcome in terms of cognitive disorders potentially associated with substance use, making it difficult to identify dissociative symptomatology when it occurs. However, we could also consider the use of substances and the objectives sought in this context, particularly regarding emotional dysregulation, which can be identified both in this personality disorder and in substance users. Finally, Baudin et al. (2022) noted that higher dissociative experiences among study participants were associated with more severe SUD. While most studies acknowledged a positive link between dissociation and SUD, a minority could not demonstrate a significant correlation.

Finally, four studies in this review highlighted a mediating effect of dissociation on the link between adverse childhood experience and substance use (Boughner & Frewen, 2016; Klanecky et al., 2016; Patel et al., 2022; Schimmenti et al., 2022). In the study by Chana et al. (2021), this result was discussed in terms of confusing the subjective experience of dissociative symptoms and substance use: in some cases, the experience of taking drugs can be like the experience of dissociation. The authors therefore recommend that future studies include a qualitative interview to address this limitation when administering dissociation questionnaires.

Studies by Baudin et al. (2022), Blanco et al. (2020), Evren et al. (2017) and Mergler et al. (2017) highlighted the high prevalence of anxiety and depression-type comorbidities among subjects with traumatic childhood experiences, SUD and dissociative experiences.

### General Trend in Studies and Consideration of Heterogeneous Data

Most of the studies included in this review focused on clinical populations (*n* = 9), particularly patients hospitalized for substance use or PTSD. The general trend revealed by these studies is that traumatic childhood experiences are associated with more severe dissociative symptoms and other severe comorbidities (traumatic, anxiety and depressive symptoms), compared to those without aversive childhood experiences (Baudin et al., 2022; Blanco et al., 2020; Boughner & Frewen, 2016; Patel et al., 2022; Somer et al., 2019). Only Mergler and collaborators (2017) did not find these results in the population treated for SUD: when comparing a group of patients with dissociative symptoms and those without: there was no difference in alcohol consumption or in the history of childhood trauma (although the questionnaires used were the same as those used in previous studies). In short, whether or not the dissociative mechanism was present, or whether or not a traumatic history was present, the proportion of drug or alcohol use was the same. However, these results seem logical given the population studied, i.e. patients in a SUD treatment center, who therefore have an addiction deemed severe enough to benefit from follow-up in a specialized center.

This general tendency for our three variables to be intertwined is also evident in the prison population (Altintas & Bilici, 2018; Chana et al., 2021), particularly when SUD and dissociative experiences are associated with traumatic childhood experiences. More surprisingly, the same pattern of results has been found in general populations where traumatic childhood events can be identified (Boughner & Frewen, 2016; Daisy & Hien, 2014), especially in the university student population (Hoktem et al., 2021; Klanecky et al., 2016). However, studies among students focused on alcohol consumption only and not on drug use. The only study focusing on a general population and drug use (Ó Laoide et al., 2018) reports that depersonalization is closely linked to drug use, including cocaine, ecstasy, heroin or cannabis, and non-prescription drugs such as benzodiazepines. Depersonalization is identified as a marker of a childhood history of emotional and physical neglect (Schimmenti et al., 2022).

Identifying the different types of traumatic childhood events experienced by subjects in each study is challenging. The extensive use of questionnaires such as the Childhood Trauma Questionnaire (CTQ) allows us to consider emotional and physical neglect, as well as emotional, physical and sexual abuse. Physical abuse in childhood appears to be one of the most significant predictors of dual pathology (Somer et al., 2019). Additionally, sexual violence in childhood increases the likelihood of re-exposure to sexual and physical violence in adulthood, presenting a potential risk of revictimization according to the study of Young et al. (2017). However, these results must be interpreted with caution, as this study focuses on a population of homeless women, whose precarious situation may increase their risk of experiencing violence.

## Discussion

This systematic review is the first to examine the impact of dissociative experiences on substance use in individuals with a history of childhood trauma. Most of included studies support a positive correlation with substance use, suggesting that greater dissociative experiences are associated with more severe substance use. Articles that did not report these positive correlations showed weak and non-significant results. Four studies indicate that dissociation acts as a mediator between substance use and the severity of traumatic symptoms. This mediation is supported by the adaptive role of substance use as a coping strategy, which helps suppress intrusive symptoms related to the traumatic experience (hence the clarification of the contribution of self-medication theory) and influences the association between trauma exposure and substance use (Boughner & Frewen, 2016). The severity of traumatic symptoms is associated with an increase in dissociation symptoms, which in turn are linked to greater alcohol or substance use problems (Patel et al., 2022). According to Schäfer and colleagues (2010), studies that do not find a mediation between dissociation, trauma and substance use focused solely on physical and sexual abuse, neglecting other forms of trauma, like emotional abuse. It is therefore crucial to consider the nature of the traumatic event. The studies conducted on general populations, university populations or clinical population with PTSD, but did not specifically focus on population with SUD.

These findings were discussed in relation to the *self-medication* and the *chemical dissociation* hypotheses, raising questions about the causal link between substance misuse, dissociative experiences and traumatic events in childhood.

The self-medication theory posits that substance use regulates post-traumatic symptoms (Khantzian, 1997), without considering dissociation. Conversely, the chemical dissociation theory suggests that substance use maintains the dissociative process. Dissociation serves as a coping strategy, enabling individuals to psychologically survive traumatic events and intense emotional reactions they have not learned to manage (Lam et al., 2024). When dissociative experiences are insufficient, substances fill the gap.

Therefore, the self-medication and chemical dissociation hypotheses are not mutually exclusive: Hoktem et al. (2021), Klanecky et al. (2016) and Güleç et al. (2014) explored the intertwining of these hypotheses in their studies. Substance use helps cope with trauma by including chemical dissociation when dissociative capacities are inadequate and alleviating post-traumatic symptoms, notably by reducing negative affects such as anxiety and depression. In our view, the *chemical dissociation theory* extends the logic of self-medication.

The intertwining of SUD and dissociative experiences in adults who have experienced childhood trauma raises questions and opens up a field that needs further exploration. Articles not included in this systematic review often had the same issue: they focused solely on the relationship between C-PTSD and substance misuse, or C-PTSD and dissociative experiences. In fact, analysis of the psychological processes involved in complex trauma often fails to identify relationships between SUD and dissociation. This review demonstrated the importance of identifying this under-researched link. Other studies have focused on different clinical samples, not presenting traumatic events in childhood but showing results consistent with this review. For example, in the study by Tsai et al. (2015), the authors focused on US veterans with wartime PTSD, with or without dissociative symptoms. The dissociative PTSD subgroup presented more severe traumatic symptoms, comorbid anxiety and depressive symptomatology, as well as more problematic alcohol use than the non-dissociative PTSD subgroup. This highlighted that the positive correlations between dissociative experiences and substance misuse can be maintained in other cases of complex trauma. Future research would be interesting to analyze the possibility of this link in other types of traumatic events.

The study by Young et al. (2017) is unique in presenting results according to each drug listed in the sample. For instance, the authors note that stimulant use can be predicted by sexual violence experienced within six months, and that amphetamine use significantly increases the odds of being a victim of physical violence. Other studies listing the drugs used by participants only address substance use disorder in general. It would be beneficial for future studies to mention results according to the specific drug consumed, as different drugs have varying psychological effects.

### Limitations

This systematic review of the literature had some limitations, both in its conception and in the original studies included.

This review focuses on a specific population, namely adults who have experienced traumatic events in childhood. Consequently, despite a rigorous and transparent research procedure, some research may not have been included because it does not specify the age at which the traumatic event occurred. Additionally, the heterogeneous results observed may be due to differences between tools for measuring dissociation and substance use. Due to this heterogeneity, we were unable to conduct a meta-analysis, despite controlling for moderating variables. Then, all the articles included in this review were based on self-reports, which may introduce classic social desirability or symptom minimization biases. The studies also varied in terms of socio-demographic characteristics of the sample. Finally, the majority of studies included in this review were cross-sectional, with only being longitudinal. Therefore, the results may reflect individual dispositions associated with a one-session assessment rather than an evolutionary process. Finally, dissociation has multiple assessment tools available, resulting an heterogeneity of assessment modalities. Dissociative symptomatology refers to a continuum ranging from everyday experiences of dissociation to dissociative identity disorder. The various measures of dissociation presented in this review addressed dissociative experiences in subjects' daily lives, although the Cambridge Depersonalization Scale (CDS) refers only to depersonalization and not to derealization. The exclusion of studies dealing with dissociative identity disorder allowed us to focus on dissociative symptoms from peritraumatic dissociation, rather than dissociation as encountered in dissociative identity disorder. A challenge for future research will be to homogenize the different assessments of complex trauma and dissociation, to make research results comparable with each other.

This literature review has highlighted the importance of examining the relationship between dissociation and substance use disorder in adults with a history of childhood trauma. It enhances our understanding of the comorbid relationship between these three clinical entities—trauma, dissociation, and substance use disorders—particularly regarding the role of substances as moderators to induce a dissociative state and mitigate traumatic symptomatology. This paper clarifies the relationship between dissociation and substance use in a population traumatized in childhood, a link seldom addressed in current literature, despite its crucial importance in psychotherapeutic treatment.

The strength of this review lies in the integration of diverse samples, ranging from the general population to hospitalized patients, allowing for a broader generalization of the subject. Future research should aim to confirm the theoretical model of substance misuse as both a means of self-medication and a reinforcing or maintenance of chemical dissociation. Moreover, it is essential to clarify the nature of the relationship between dissociation, substance misuse and traumatic symptoms. The psychological effects of substances may fill a gap in dissociative processes or enable the re-experiencing of dissociative experiences. This understanding will help clinicians develop models to conceptualize these relationships and derive management strategies that consider the defensive function of substance use.

### Implications for Practice and Research

The literature already addresses the vicious circle maintained by problematic substance use and traumatic symptomatology, particularly with the term dual pathology. However, the impact of dissociation within this cycle is often overlooked. The severity of SUD is exacerbated when dissociative symptomatology is present, which must therefore become a point of vigilance for therapists (Caretti et al., 2018). Dissociation not only aggravates traumatic symptomatology but also strengthens our understanding of the addictive profile, which is now related to maladaptive functioning such as dissociation, emotional dysregulation, obsessive thoughts or separation anxiety (Caretti et al., 2018). Moreover, SUD may influence the persistence and severity of dissociative symptomatology (Wegen et al., 2017). These dissociative experiences are particularly present during acute withdrawal; dissociation and traumatic symptomatology being reported as higher from the first weeks of abstinence, particularly from alcohol and cocaine (Coffey et al., 2007). Dissociation is therefore an important point to consider during treatment, particularly through integrative care. In subjects with SUD, dissociation followed by improvement in affect regulation skills could prevent relapse (Wegen et al., 2017). However, future research will need to focus on these complex treatments, while psychotherapeutic treatments for comorbid disorders in dual pathology (PTSD and SUD) are still under consideration and constitute a major public health issue.

## Supplementary Materials

The Supplementary Materials contain the excel of eligible articles (Julien et al., 2024S).



JulienC.
BernardL.
BrejardV.
 (2024S). Dissociative experiences and addictions in adulthood after childhood trauma: A systematic review of the literature. Raw data CJ LB VB 2024.xlsx
[Research data]. PsychOpen. https://osf.io/526rx
10.32872/cpe.15877PMC1292318941725696

## Data Availability

The excel of eligible articles is available at the OSF (Julien et al., 2024S).

## Preregistration

Preregistration was not performed.

## Social Media Accounts

*Cory Julien:* LinkedIn

## Reporting Guidelines

This article was prepared following the PRISMA guidelines.
